# Asymptomatic young male with ectopia cordis interna: A rare case report: Retraction

**DOI:** 10.1097/MD.0000000000044334

**Published:** 2025-08-22

**Authors:** Hashim Talib Hashim, Mohammedbaqer Ghuraibawi, Fatimah Abdullah Sulaiman, Batool S. Al-Aboudi, Zainab Ahmed Lateef, Ashraf Fhed Mohammed Basalilah, Bashar Hadi Shalan

The article “Asymptomatic young male with ectopia cordis interna: A rare case report”^1^, which appears in Volume 104, Issue 30 of *Medicine*, is being retracted. After publication, a concerned reader contacted the Editorial Office alleging that the images in the case appeared to be direct reproductions from a satirical online publication.^2^ The authors were asked to provide supporting documentation for their case in response, including the original radiographic image files in DICOM format, and they were unable to provide the necessary documentation, which casts doubt on the credibility of the presented case. Upon further investigation, additional concerns were raised regarding the authorship, and the Publisher no longer has confidence in the integrity of the content or authorship.
